# Predicting COVID-19–Related Health Care Resource Utilization Across a Statewide Patient Population: Model Development Study

**DOI:** 10.2196/31337

**Published:** 2021-11-15

**Authors:** Suranga N Kasturi, Jeremy Park, David Wild, Babar Khan, David A Haggstrom, Shaun Grannis

**Affiliations:** 1 Regenstrief Institute Indianapolis, IN United States; 2 Department of Pediatrics Indiana University School of Medicine Indianapolis, IN United States; 3 Luddy School of Informatics Computing and Engineering Indiana University Bloomington, IN United States; 4 Department of Medicine Indiana University School of Medicine Indianapolis, IN United States; 5 Department of Family Medicine Indiana University School of Medicine Indianapolis, IN United States

**Keywords:** COVID-19, machine learning, population health, health care utilization, health disparities, health information, epidemiology, public health, digital health, health data, pandemic, decision models, health informatics, healthcare resources

## Abstract

**Background:**

The COVID-19 pandemic has highlighted the inability of health systems to leverage existing system infrastructure in order to rapidly develop and apply broad analytical tools that could inform state- and national-level policymaking, as well as patient care delivery in hospital settings. The COVID-19 pandemic has also led to highlighted systemic disparities in health outcomes and access to care based on race or ethnicity, gender, income-level, and urban-rural divide. Although the United States seems to be recovering from the COVID-19 pandemic owing to widespread vaccination efforts and increased public awareness, there is an urgent need to address the aforementioned challenges.

**Objective:**

This study aims to inform the feasibility of leveraging broad, statewide datasets for population health–driven decision-making by developing robust analytical models that predict COVID-19–related health care resource utilization across patients served by Indiana’s statewide Health Information Exchange.

**Methods:**

We leveraged comprehensive datasets obtained from the Indiana Network for Patient Care to train decision forest-based models that can predict patient-level need of health care resource utilization. To assess these models for potential biases, we tested model performance against subpopulations stratified by age, race or ethnicity, gender, and residence (urban vs rural).

**Results:**

For model development, we identified a cohort of 96,026 patients from across 957 zip codes in Indiana, United States. We trained the decision models that predicted health care resource utilization by using approximately 100 of the most impactful features from a total of 1172 features created. Each model and stratified subpopulation under test reported precision scores >70%, accuracy and area under the receiver operating curve scores >80%, and sensitivity scores approximately >90%. We noted statistically significant variations in model performance across stratified subpopulations identified by age, race or ethnicity, gender, and residence (urban vs rural).

**Conclusions:**

This study presents the possibility of developing decision models capable of predicting patient-level health care resource utilization across a broad, statewide region with considerable predictive performance. However, our models present statistically significant variations in performance across stratified subpopulations of interest. Further efforts are necessary to identify root causes of these biases and to rectify them.

## Introduction

### Background

The COVID-19 pandemic has impacted the health and well-being of individuals, communities, and economies worldwide at an unprecedented scale [1,2]. As of June 1, 2021, the COVID-19 pandemic has infected over 170 million people worldwide and claimed the lives of over 3.5 million people. In the United States alone, COVID-19 has infected over 33 million people and claimed over 600,000 lives. In addition to the loss of lives and other adverse health outcomes, the enforcement of preventative measures, such as lockdowns and mask-wearing mandates, have further affected the mental and physical well-being of individuals and communities. The cumulative financial costs of the COVID-19 pandemic caused by lost output and health reduction has been estimated at US $16 trillion, or approximately 90% of the annual gross domestic product of the United States [3].

In the United States, the COVID-19 pandemic has highlighted (1) the inability of health systems to leverage existing system infrastructure in order to rapidly develop and apply broad analytical tools that could inform state- and national-level policymaking and patient care delivery in hospital settings and (2) systemic disparities in COVID-19–related outcomes and access to care based on race or ethnicity [4], gender [5], income level, and urban-rural divide [6,7]. At the peak of the pandemic outbreak in the United States, these limitations contributed to distrust, misinformation, and lack of cohesive decision-making. This impeded local government and public health officials from making informed policy decisions, such as mask-wearing mandates and stay-at-home orders, to control disease outbreaks and safeguard health systems from extended strain. This led to shortages in hospital beds, personal protective equipment, and health care personnel, thereby causing significant disruptions to health care delivery and consequent loss of lives [2,3].

Although the United States seems to be recovering from the COVID-19 pandemic owing to widespread vaccination efforts and increased public awareness, there is still a need to address the aforementioned limitations. Overcoming these limitations will ensure better disaster preparedness and response in anticipation of any future outbreaks caused by either COVID-19 variants or other diseases and to manage the care of vaccine-hesitant populations. The United States boasts significant health information system infrastructure, resulting in the active collection of a wide variety of patient-level clinical, medication, and visit history data. However, such datasets are often siloed across different health systems. As a result, analytical model development is often spearheaded at the health system level. Although such models may be useful in caring for a specific health system, they may not generalize across broader populations and cannot contribute to large-scale public health responses delivered across broad geographies, such as at the county, metropolitan area, or state level.

### Objective

In this study, we sought to inform the feasibility of leveraging broad, statewide datasets for population health–driven decision-making by developing robust analytical models that predicted COVID-19–related health care resource utilization at the patient level among those served by Indiana’s statewide Health Information Exchange (HIE).

## Methods

### Patient Population and Data Sources

We leveraged the COVID-19 Research Data Commons (CoRDaCo) [8], a rich, statewide dataset curated by the Regenstrief Institute of Indianapolis and Indiana University. The CoRDaCo dataset seeks to enable better access to data on COVID-19–positive patients for research purposes. It integrates data from multiple clinical sources, including the Indiana Network for Patient Care (INPC) [9]—one of the longest continuously operated statewide HIEs in the United States consisting of data from over 15 million inhabitants of Indiana spread across 23 health systems and 93 hospitals, as well as other state laboratory reporting state vitals data. The INPC patient population represents a variety of health systems spread across Indiana [10] (representation of COVID-19 patient dataset is illustrated in detail in the Results section). This is relevant given that Indiana is representative of the total US population in terms of age, gender, education levels [11] and urban-rural divide [12]. For each patient, CoRDaCo includes data captured between January 1, 2018, and November 30, 2020. The data pull was performed by specialized analysts from the Regenstrief Institute Data Core—the only personnel permitted direct access to identifiable patient data within the INPC research database.

### Preparation of Feature Sets

We extracted and vectorized a wide variety of patient-level features representing their demographics; diagnoses; past encounter history; medications; and social determinants of health, defined as conditions in which people are born, grow, live, work and age [13] (Table 1).

Creation of feature vectors for model development was performed by the authors using the python programming language.

**Table 1:** List of features extracted for model development.

Data type	Description of features modeled
Demographics	Patient age, gender, race or ethnicity represented as integer and categorical variables
Diagnosis data	Represented as integer variables: Charlson comorbidity index [14] Represented as a Boolean values: Presence of most commonly occurring chronic conditions [15] Diagnoses of addictions, behaviors, behavioral disorders, and narcotics use [16] Presence of 1000 most frequently reported diagnoses identified using the International Classification of Diseases
Past encounter history	Inpatient, outpatient, and emergency visits represented as counts
Medications	Medications categorized into diagnosis groups and represented as Boolean values
Social determinants of health	Represented as a Boolean values: Socioeconomic status (unemployment, type of insurance) Education Neighborhood and physical environment Urban vs rural status classified using Rural-Urban commuting area (RUCA) codes [17] Employment Social support networks Access to health care according to the Kaiser Family Foundation framework [18] All features were inferred using patient-level diagnosis codes and patient address information.

### Development of a Gold Standard

We parsed past encounter history data on each patient to identify those who had been hospitalized (defined as patients who had been admitted to either inpatient or intensive care) within either of the following:

- The first week of receiving a diagnosis of COVID-19 (ie, 1-week cohort), including a measure of which patients were in need of urgent care at the time of, or soon after, diagnosis.
- The first 6 weeks of receiving a diagnosis of COVID-19 (ie, 6-week cohort). A metric of which patients would need inpatient care during the course of their illness [19].

To ensure that our gold standard focused on inpatient or intensive care unit stays influenced by COVID-19 alone, we applied regular expressions to patient admission reason notes in order to identify and exclude any admissions due to accidents such as falls, injuries, lacerations, and fractures, as well as suicidal ideation, overdoses, and alcohol abuse. These factors were selected for exclusion based on an assessment of the most frequently occurring admission reasons identified from patient hospitalization datasets.

**Figure 1:**
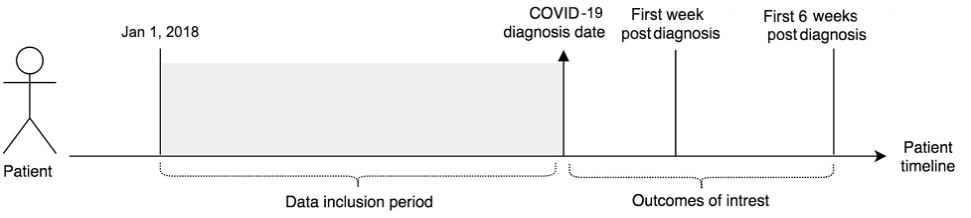
Feature vector preparation and detection of outcomes of interest based on the patient’s longitudinal health history.

Figure 1 represents our approach to feature vector preparation and detection of outcomes of interest for analytical modelling based on the patient’s longitudinal health history.

### Machine Learning Process

We leveraged Python and the scikit-learn machine learning library [20] to train prediction models using the eXtreme Gradient Boosting (XGBoost) algorithm [21]. The XGBoost algorithm is an implementation of gradient-boosted ensemble decision trees [22] designed to optimize speed and performance. XGBoost classification was selected because research conducted by ourselves, as well as other external groups found that ensemble decision trees performed compatibly, or better than other classification algorithms [23,24] and because XGBoost could be trained using a smaller number of features than those required to train neural networks and other deep learning–based models, which enables ease of model development, interpretability, and explainability. We split each data vector into random groups of 80% (training and validation dataset) and 20% (holdout test set). We then leveraged the 80% training and validation dataset to train optimal models for each scenario by using 10-fold crossvalidation and hyperparameter turning and methods. To enable better generalization of each model, we applied the internal feature selection method of XGBoost [25], which prioritizes feature importance based on average gain across all splits the feature is used in, to restrict models to a smaller subset of the most relevant features.

### Model Evaluation

We assessed the performance of each decision model in the 20% holdout test dataset by using several performance metrics:

- Positive predictive value, or *precision*: the likelihood that a positively identified case is truly positive.
- Sensitivity, or *recall*: the likelihood that a true positive case is correctly identified as positive.
- Specificity: the likelihood that a negative case is correctly identified as negative.
- F_1_ score: the harmonic mean of model precision and recall scores.
- Accuracy: the likelihood that a prediction is correct.
- Area under the receiver operating curve (AUC-ROC): a metric representing the performance of a prediction model at all classification thresholds.

### Evaluation of Analytical Performance Against Subpopulations

**Figure 2:**
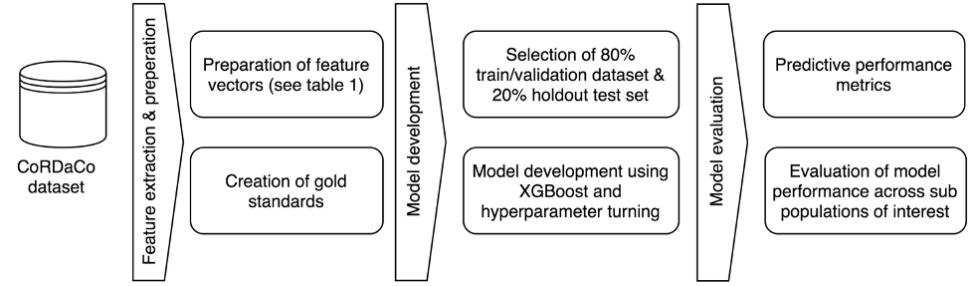
Workflow presenting the complete study approach from data extraction to predictive model evaluation. CoRDaCo: COVID-19 Research Data Commons.

As discussed previously, the COVID-19 pandemic has highlighted systemic disparities in patient outcomes and access to care based on race or ethnicity [4], gender [5], income level, and urban-rural divide [6,7]. These disparities may be present in the datasets used to train analytical models, resulting in biased predictions that place privileged groups at a systematic advantage and unprivileged groups at a systematic disadvantage [26]. To evaluate our models for such biases, we stratified the holdout test dataset by age, race or ethnicity, gender, and residence (urban vs rural), and we evaluated model performance across each stratified subpopulation by using the same performance metrics. Figure 2 provides a comprehensive overview of our study approach.

### Human Participants Research Approval

This study was approved by the Indiana University’s Institutional Review Board (2005573466).

## Results

### Overview

The CoRDaCo dataset consisted of 230,981 patients with a positive COVID-19 diagnosis. However, we noted that a considerable number of these patients were out-of-state residents who visited health systems that were part of the INPC only to obtain COVID-19 tests or were Indiana residents whose only interaction with INPC-affiliated health systems were to undergo COVID-19 testing. As such, we had no clinical data beyond COVID-19 status on these patients. To enrich the quality of datasets used for model building, we excluded such patients by identifying and removing any patient whose only INPC record was a positive COVID-19 test result. This resulted in a total of 96,115 patients. We excluded an additional 89 patients owing to errors in their medical records, resulting in a total of 96,026 *legacy patients* to be included in our model development efforts. This legacy population was from a diverse race or ethnicity (27% Black, Hispanic, and others), predominantly adult (median age 47 years [33.73]), mostly urban (76,988/96,026, 80.17%), and had a larger representation of females (57,475/96,026, 59.85%). A total of 18,694 (19.47%) of these patients were hospitalized during the first week of being diagnosed with COVID-19, whereas 22,678 (23.62%) were hospitalized during the first 6 weeks of receiving a COVID-19 diagnosis.

**Table 2:** Characteristics of the patient populations used for analytical model development.

Patient characteristics		COVID-19 patient cohort	Patients hospitalized during the first week	Patients hospitalized during the first 6 weeks
**Gender, n (%)**				
	Male	38,529 (40.12)	8178 (43.75)	9615 (42.40)
	Female	57,475 (59.85)	10,516 (56.25)	13,062 (57.60)
	Unknown	22 (0.02)	0 (0)	1 (0)
**Race or ethnicity, n (%)**				
	White, non-Hispanic	70,238 (73.15)	11,783 (63.03)	14,737 (64.98)
	Black, non-Hispanic	12,372 (12.88)	4,104 (21.95)	4666 (20.58)
	Hispanic	9882 (10.29)	2171 (11.61)	2,533 (11.17)
	Other	3534 (3.68)	636 (3.40)	742 (3.27)
**Age (years), n (%)^a^**				
	Minors (<18 years)	7064 (7.36)	638 (3.41)	754 (3.34)
	Adults (18-65 years)	67,563 (70.36)	11,330 (60.61)	13,851 (61.08)
	Older adults (>65 years)	21,177 (22.05)	6726 (35.98)	8074 (35.60)
	Unknown	222 (0.23)	0 (0)	0 (0)
**Residence, n (%)**				
	Number of zip codes represented	957 (99.90)	678 (70.85)	705 (73.67)
	Living in an urban area	76,988 (80.17)	14,833 (79.35)	17,910 (78.98)
	Living in a rural area	16,843 (17.54)	3267 (17.48)	4084 (18.01)
	Unknown	2195 (2.29)	594 (3.18)	684 (3.02)
**Encounters, mean (SD)**				
	Outpatient visits	7.715 (10.09)	9.391 (13.18)	9.530 (12.29)
	Emergency room visits	0.926 (2.25)	2.431 (3.52)	2.237 (3.45)
	Hospitalizations	0.339 (1.35)	0.938 (2.24)	0.875 (2.19)
**Chronic disease burden, n (%)**				
	Cancer	3976 (4.14)	1226 (6.56)	1484 (6.54)
	Diabetes with complications	4340 (4.52)	1903 (10.18)	2222 (9.80)
	Diabetes without complications	10,819 (11.27)	3845 (20.57)	4506 (19.87)
	Dementia	2529 (2.63)	648 (3.47)	871 (3.84)
	Chronic pulmonary disease	10,755 (11.20)	2364 (12.65)	4338 (19.13)
	Renal disease	5449 (5.67)	2397 (12.82)	2794 (12.32)

^a^Mean participant age: 47.039 years (21.43).

### Model Development and Evaluation

**Figure 3:**
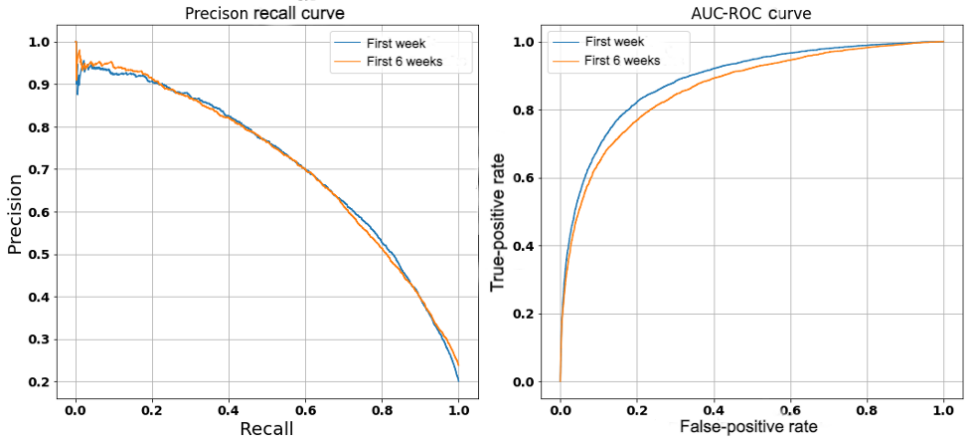
Precision-recall and AUC-ROC: area under the receiver operating curve (AUC-ROC) curves for each prediction model.

The feature preparation process (Table 1) resulted in a total of 1172 features for model training. To enable model generalizability and ease of interpretation, we restricted each model to approximately the most significant 100 features selected based on feature importance threshold drop-offs. Table 3 presents performance metrics reported by each model across the 20% holdout test dataset. Figure 3 presents the precision-recall and AUC-ROC curves for each prediction model. The subset of features included in each model is presented in Multimedia Appendix 1.

Both models delivered strong performance metrics. However, the model for the 1-week cohort reported significantly greater specificity, accuracy, and AUC-ROC scores than the 6-week cohort model.

**Table 3:** Predictive model performance.

Performance metric	First week (95% CI)	First 6 weeks (95% CI)
Precision	75.133 (73.445-76.822)	73.697 (72.142-75.253)
Sensitivity	52.505 (50.875-54.136)	52.571 (51.081-54.061)
Specificity	95.780 (95.457-96.104)	94.269 (93.887-94.653)
Accuracy	87.326 (86.846-87.806)	84.514 (83.992-85.037)
AUC-ROC^a^	88.744 (88.136-89.205)	86.215 (85.773-87.091)
F_1_ score	61.814 (60.092-63.535)	61.367 (59.797-62.936)

^a^AUC-ROC: area under the receiver operating curve.

### Evaluation of Analytical Performance Against Subpopulations

To assess model performance across different subpopulations of interest, we stratified the holdout test dataset by age, race or ethnicity, gender, and residence (urban vs rural), and we then evaluated their performance using each performance metric. Tables 4 and 5 present statistically significant variations in predictive performance reported across each model. Comprehensive predictive performance metrics, together with 95% CIs are listed in Multimedia Appendix 2. AUC-ROC curves for the performance of models across each stratified subpopulation are presented in Multimedia Appendix 3.

**Table 4:** Statistically significant performance variations in model to predict health care resource utilization within the first week.

Performance metric	Urban vs rural	Male vs female	Minors vs adults vs older adults	White vs Black vs Hispanic
Precision	No difference	No difference	No difference	No difference
Sensitivity or recall	Urban > rural	Male > female	Older adults > (adults = minors)	Black > Hispanic > White
Specificity	No difference	No difference	Minors > adults > older adults	(White and Hispanic) > black
Accuracy	No difference	No difference	Minors > adults > older adults	(White and Hispanic) > black
AUC-ROC^a^	No difference	Male > female	Minors > adults > older adults	No difference
F_1_ score	Urban > rural	Male > female	(Older adults = minors) > adults	(Black and Hispanic) > White

^a^AUC-ROC: area under the receiver operating curve.

**Table 5:** Statistically significant performance variations in model to predict health care resource utilization within the first 6 weeks.

Performance metric	Urban vs rural	Male vs female	Minors vs adults vs older adults	White vs Black vs Hispanic
Precision	No difference	No difference	No difference	No difference
Sensitivity or recall	Urban > rural	Male > female	Older adults > (adults = minors)	Black > Hispanic > White
Specificity	No difference	No difference	Minors > adult > senior	White & Hispanic > black
Accuracy	No difference	No difference	Minors > adult > senior	No difference
AUC-ROC^a^	Urban > rural	Male > female	Minors > adult > senior	No difference
F_1_ score	Urban > rural	Male > female	(Older adults = minors) > adults	Black > Hispanic > White

^a^AUC-ROC: area under the receiver operating curve.

As presented in Tables 4 and 5, there were no statistically significant differences in precision scores reported across each strata or model under test. However, we found evidence of significant variations in model performance across many other strata. Across both models and all performance metrics under test, residing in an urban area was associated with comparable, or higher predictive performance than if residing in a rural area. Across both models and all performance metrics under test, being male was associated with comparable, or higher predictive performance than if female. Performance stratified by age showed significant variations, with some performance metrics favoring older adults while others favored minors. These results are indicative of biases learned from underlying data sources used for model development, or inefficient learning parameters implemented by the machine learning algorithm.

## Discussion

### Principal Findings

Our results demonstrate the ability to train decision models capable of predicting the need of COVID-19–related hospitalization across a broad, statewide patient population with considerable performance accuracy. The 1-week model for predicting the need of COVID-19–related hospitalization reported specificity, accuracy, and AUC-ROC scores that were significantly larger than the 6-week model. The findings are intuitive given that hospitalization risk is more predictable over shorter time frames. Such utilization prediction models may be used for population health management programs in health systems, to identify high-risk populations to monitor or screen, as well as predicting resource need in crisis situations, such as future spikes in pandemic activity or outbreaks.

Stratification of model performance across age, race or ethnicity, gender, and urban versus rural divide identified statistically significant variations in model performance across subpopulations. Each model and stratified subpopulation under test reported precision scores >70%, accuracy and AUC-ROC scores >80%, and sensitivity scores approximately >90%. We note that recall scores for each model (approximately 50%-54%) were lower than ideal, implying that a considerable proportion of patients in need of health care services were being ignored. However, model precision, which is indicative of what percentage of patients identified by the model actually needed care was high (>70%), suggesting that it was pragmatic for use in clinical settings. Additionally, model specificity scores were very high (approximately >90%). This finding indicated that the models were able to correctly identify patients who were not in need of care with very high accuracy, which is very valuable in making clinical decisions on which patients to prioritize.

Features that influenced the prediction of health care resource utilization included patient age [27], chronic obstructive pulmonary disease status [28], smoking [28], diabetes [29], indication of neurological diseases via diagnosis (eg, dementia [30]) or medications (eg, anti-Parkinson and related therapy agents), mental disorders (eg, anxiety disorders), residence (urban vs rural) [31,32], and income-level, measured on the basis of the type of insurance used by the patient. None of the patient-level social determinants of health factors extracted from the International Classification of Diseases diagnosis data were found to be impactful enough for inclusion in either model. This could be attributed to the scarcity of these elements being captured in clinical settings. However, patient-level features on the type of insurance (which is indicative of an individual’s financial and employment status) and RUCA code (which could be used to infer an individual’s income level, isolation, and access to services and health resources) were both widely available. These elements were found to be impactful and were integrated into both models.

Each model exhibited significant variations in predictive performance across subpopulations. Overall, male gender or living in an urban area was associated with stronger predictive performance. These differences may be influenced by variations in access to health care services or health care delivery prevalent in the datasets, and the models could learn them during the training process. We cannot make further assumptions on the causes of varying model predictions without a proper assessment of underlying causes of this behavior.

### Limitations

We noted several limitations to this study. We leveraged statewide datasets from the INPC HIE system to ensure that our models could be operationalized across a broad geographic region. As such, our modeling did not include data elements that were collected by health systems but not shared with the INPC. Since the collection of such datasets and their availability at the HIE level may vary based on the health system, the inclusion of such elements may impact the generalizability of our models across different health systems. Our use cases assessed the need of hospitalization during the first 6 weeks of diagnosis. This excludes the needs of patients suffering long-COVID, where patients may not fully recover for several months [33]. Models were trained using *legacy patients*, who were participants of the INPC system prior to March 1, 2020. It is unclear how the models will perform against other patients who do not regularly interact with the health system and sought care only for COVID-19 testing purposes. This is concerning given that such patients may suffer from a higher disease burden. Our modelling efforts covered a broad time period spanning several waves of the COVID-19 pandemic, as well as the enforcement and relaxation of various mandates aimed at controlling COVID-19 infection rates. These changes may have influenced the capacity of hospital systems resulting in changes in how many patients were provided inpatient care. Alternatively, hospital admission and emergency management protocols may have also changed throughout this period, further impacting which patients received care. Our current effort did not consider how these variations influence the training datasets, and as such, how our models would generalize across future outbreaks and mandates, as COVID-19 infection rates continue to change. Future research will systematically investigate and calibrate model performance across different stages of the pandemic.

We sought to demonstrate the ability to develop broad, state-level models for COVID-19–related research. As such, the biases in analytical models detected in this study highlight significant concerns that researchers must protect against. These biases in analytical model performance will be addressed during the next phase of our work. Further, although the generalizability of our models across other states is untested, they can influence other emerging COVID-19 analytical efforts. In particular, these models can influence data collection, curation, and modeling activities undertaken by the National COVID Cohort Collaborative (N3C) [34], which is stewarded by the National Center for Advancing Translational Sciences and hosts data on over 250,000 COVID-19–positive patients from 31 sites spread across the United States. N3C could serve as an in-vivo laboratory for our research efforts.

### Conclusions

This study presents the possibility of developing decision models capable of predicting patient-level health care resource utilization across a broad, statewide region with considerable predictive performance. However, the analytical models present statistically significant variations in performance across stratified subpopulations of interest. Further efforts are necessary to identify root causes of these biases and to rectify them.

## Appendix

Multimedia Appendix 1 List of top-ranking features included in each predictive model.

Multimedia Appendix 2 Performance metrics reported by each analytical model across each stratified subpopulation of the study.

Multimedia Appendix 3 Area under the receiver operating curves (AUC-ROCs) for the performance of models across each stratified subpopulation.

## Abbreviations

AUC-ROC: Area under the receiver operating curve
CoRDaCo: COVID-19 Research Data Commons
HIE: Health Information Exchange
INPC: Indiana Network for Patient Care
N3C: National COVID Cohort Collaborative
RUCA: Rural-Urban commuting area
XGBoost: eXtreme Gradient Boosting
