# Supplementary material for: Predicting COVID-19–Related Health Care Resource Utilization Across a Statewide Patient Population: Model Development Study
Source: J Med Internet Res. 2021 Nov 15;23(11):e31337. doi: 10.2196/31337 (PMC8594735; doi:10.2196/31337)
Supplement: Multimedia Appendix 1 [file jmir_v23i11e31337_app1.docx]

Appendix 1. List of top-ranking features included in each predictive model.

| Need of hospitalization within 1^st^ week of positive COVID-19 test |
| --- |
| Demographics: Age, ethnicity, race, gender  Residence: Census tract, zip code, RUCA categories  Insurance: Type of insurances used; commercial, Medicare, Medicaid or self-pay.  Past encounters: Past history of inpatient, outpatient and emergency department visits.  Diagnosis: Other general symptoms and signs, Viral infection, Pneumonia, Shortness of breath, Unspecified abdominal pain, Chronic pulmonary disease, Encounter for general adult medical examination without abnormal findings, primary hypertension, Fever, Other specified counseling, morbid obesity, Encounter for other preprocedural examination, other long term (current) drug therapy, chest pain, Acute upper respiratory infection, Hypoxemia, Cough, Other viral pneumonia, Dementia, other, nonpsychotic mental disorders Hyperlipidemia, Encounter for screening mammogram for malignant neoplasm of breast, Acute respiratory failure with hypoxia, Encounter for immunization, Renal disease, Urinary tract infection, headache  Medications: Analgesics-Opioid, Corticosteroids, Antihypertensives, Antidepressants, Antidiabetics, Antihyperlipidemics, Antiasthmatic And Bronchodilator Agents, Anti-Infective Agents-Misc., Beta Blockers, Anticonvulsants, Analgesics-Anti-Inflammatory, Diuretics, Ulcer Drugs/Antispasmodics/Anticholinergics, Penicillins, Cephalosporins, Dermatologicals, Calcium Channel Blockers, Analgesics-Non Narcotic, Antianxiety Agents, Macrolides, Vaccines, Antifungals, Ophthalmic Agents, Nasal Agents-Systemic And Topical, Fluoroquinolones, Diagnostic Products, Cough/Cold/Allergy, Antiemetics, Laxatives, Genitourinary Agents-Miscellaneous, Musculoskeletal Therapy Agents, Anticoagulants, Minerals & Electrolytes, Gout Agents, Thyroid Agents, Hematological Agents-Misc., Tetracyclines, Psychotherapeutic And Neurological Agents-Misc., Hematopoietic Agents, Antihistamines, Antivirals, Antipsychotics/Antimanic Agents, Antiparkinson And Related Therapy Agents, Gastrointestinal Agents-Misc., Mouth/Throat/Dental Agents, Medical Devices And Supplies, Adhd/Anti-Narcolepsy/Anti-Obesity/Anorexiants, |
| Need of hospitalization within 1^st^ six weeks of positive COVID-19 test |
| Demographics: Age, ethnicity, race, gender  Residence: Census tract, zip code, RUCA score and RUCA categories  Insurance: Type of insurances used; commercial, Medicare, Medicaid or self-pay.  Past encounters: Past encounters: Past history of inpatient, outpatient and emergency department visits.  Diagnosis: Morbid obesity, Encounter for other preprocedural examination, Diabetes without complications, mood, Chest pain, Other long term (current) drug therapy, Encounter for immunization, Obesity, Hyperlipidemia, Anxiety Disorder, Nausea with vomiting, Dementia, nonpsychotic_mental, nicotine use, other, Hypothyroidism, Unspecified, Vitamin D deficiency, unspecified, Hypokalemia, Shortness of breath, Viral infection, unspecified, Fever, unspecified, Pneumonia, Primary hypertension, Hypoxemia, Other specified counseling, Encounter for general adult medical examination without abnormal findings, Other general symptoms and signs, Cough, Chronic pulmonary disease, Other viral pneumonia, Acute respiratory failure with hypoxia, Unspecified abdominal pain  Medications: Antidepressants, Analgesics-Opioid, Corticosteroids, Antihyperlipidemics, Antihypertensives, Antiasthmatic And Bronchodilator Agents, Antidiabetics, Penicillins, Anti-Infective Agents-Misc., Diuretics, Anticonvulsants, Ulcer Drugs/Antispasmodics/Anticholinergics, Analgesics-Anti-Inflammatory, Dermatologicals, Beta Blockers, Calcium Channel Blockers, Macrolides, Cephalosporins, Antianxiety Agents, Analgesics-Non Narcotic, Antifungals, Minerals & Electrolytes, Ophthalmic Agents, Genitourinary Agents-Miscellaneous, Antiemetics, Antivirals, Nasal Agents-Systemic And Topical, Fluoroquinolones, Cough/Cold/Allergy, Hematopoietic Agents, Vaccines, Musculoskeletal Therapy Agents, Hematological Agents-Misc., Tetracyclines, Laxatives, Psychotherapeutic And Neurological Agents-Misc., Thyroid Agents, Contraceptives, Antihistamines, Medical Devices And Supplies, Antipsychotics/Antimanic Agents, Gout Agents, Gastrointestinal Agents-Misc., Diagnostic Products, Vitamins, Anticoagulants, Mouth/Throat/Dental Agents, Antiparkinson and Related Therapy Agents, Adhd/Anti-Narcolepsy/Anti-Obesity/Anorexiants |
