# Supplementary material for: Predicting COVID-19–Related Health Care Resource Utilization Across a Statewide Patient Population: Model Development Study
Source: J Med Internet Res. 2021 Nov 15;23(11):e31337. doi: 10.2196/31337 (PMC8594735; doi:10.2196/31337)
Supplement: Multimedia Appendix 2 [file jmir_v23i11e31337_app2.docx]

Appendix 2. Performance metrics reported by each analytical model across each stratified subpopulation of the study.

|  | 1^st^ week (95% CI) | 1^st^ six weeks (95% CI) |
| --- | --- | --- |
| Male | | |
| Precision | 77.321 (74.921-79.722) | 76.337 (74.011-78.564) |
| Sensitivity aka. Recall | 57.553 (55.109-59.998) | 57.224 (54.980-59.467) |
| Specificity | 95.417 (94.878-95.956) | 93.959 (93.329-94.590) |
| Accuracy | 87.332 (86.572-88.092) | 84.626 (83.802-85.451) |
| AUC ROC | 89.446 (88.801-90.302) | 87.528 (86.875-88.030) |
| F1-Score | 65.989 (63.480-68.498) | 65.413 (63.107-67.719) |
| Female | | |
| Precision | 73.365 (71.046-75.685) | 71.910 (69.780-74.241) |
| Sensitivity aka. Recall | 49.306 (47.156-51.457) | 47.868 (45.936-49.800) |
| Specificity | 95.875 (95.464-96.286) | 94.364 (93.875-94.854) |
| Accuracy | 87.154 (86.531-87.776) | 83.596 (82.907-84.285) |
| AUC ROC | 87.987 (87.502-88.493) | 86.016 (85.563-86.615) |
| F1-Score | 58.976 (56.663-61.290) | 57.476 (55.381-59.572) |
| White | | |
| Precision | 73.766 (71.620-75.912) | 71.134 (69.172-73.096) |
| Sensitivity aka. Recall | 46.286 (44.359-48.213) | 45.640 (43.913-47.368) |
| Specificity | 96.525 (96.199-96.850) | 94.874 (94.473-95.277) |
| Accuracy | 87.767 (87.238-88.295) | 83.203 (82.614-84.792) |
| AUC ROC | 88.477 (87.981-89.094) | 85.916 (84.877-86.935) |
| F1-Score | 56.880 (54.759-59.002) | 55.604 (53.703-57.506) |
| Black | | |
| Precision | 75.001 (71.961-78.039) | 75.543 (72.637-78.449) |
| Sensitivity aka. Recall | 72.156 (69.071-75.242) | 71.075 (68.102-74.049) |
| Specificity | 88.190 (86.634-89.748) | 87.007 (85.350-88.664) |
| Accuracy | 82.911 (81.424-84.398) | 81.257 (79.720-82.795) |
| AUC ROC | 88.747 (87.287-90.242 ) | 86.419 (84.942-87.953) |
| F1-Score | 73.551 (70.485-76.616) | 73.241 (70.294-76.188) |
| Hispanic or Latino | | |
| Precision | 81.266 (77.258-85.275) | 76.152 (71.715-80.589) |
| Sensitivity aka. Recall | 62.729 (58.365-67.093) | 54.038 (49.667-58.409) |
| Specificity | 95.369 (94.295-96.442) | 94.026 (92.791-95.261) |
| Accuracy | 87.451 (85.978-88.923) | 83.593 (81.933-85.252) |
| AUC ROC | 90.833 (89.337-92.269) | 86.982 (85.532-88.374) |
| F1-Score | 70.805 (66.445-75.164) | 63.217 (58.643-67.792) |
| Age (<18) | | |
| Precision | 84.211 (74.551-93.870) | 82.353 (74.083-90.623) |
| Sensitivity aka. Recall | 41.379 (32.234-50.525) | 48.276 (39.976-56.575) |
| Specificity | 99.314 (98.859-99.770) | 98.911 (98.352-99.471) |
| Accuracy | 94.611 (93.417-95.806) | 94.090 (92.882-95.299) |
| AUC ROC | 93.822 (92.753-94.917) | 89.162 (88.002-90.235) |
| F1-Score | 55.491 (44.804-66.178) | 60.869 (51.768-69.972) |
| Age (>=18 and <65) | | |
| Precision | 75.284 (73.054-77.514) | 73.889 (71.805-75.973) |
| Sensitivity aka. Recall | 48.725 (46.646-50.803) | 47.504 (45.604-49.403) |
| Specificity | 96.701 (96.364-97.039) | 95.681 (95.290-96.074) |
| Accuracy | 88.499 (87.951-89.048) | 85.824 (85.224-86.424) |
| AUC ROC | 88.645 (88.167-89.190) | 85.607 (85.063-86.326) |
| F1-Score | 59.160 (56.908-61.412) | 57.829 (55.756-59.901) |
| Age (>=65) | | |
| Precision | 75.875 (73.243-78.507) | 72.897 (70.471-75.322) |
| Sensitivity aka. Recall | 58.243 (55.585-60.901) | 61.806 (59.364-64.247) |
| Specificity | 91.182 (90.128-92.237) | 85.929 (84.562-87.297) |
| Accuracy | 80.558 (79.346-81.769) | 76.768 (75.460-78.076) |
| AUC ROC | 84.522 (84.013-84.904) | 83.374 (82.604-84.101) |
| F1-Score | 65.899 (63.182-68.617) | 66.894 (64.434-69.355) |
| Urban | | |
| Precision | 73.611(71.745-75.477) | 74.118 (72.403-75.833) |
| Sensitivity aka. Recall | 54.968 (53.148-56.788) | 54.039 (52.373-55.706) |
| Specificity | 95.276 (94.896-95.656) | 94.346 (93.924-94.769) |
| Accuracy | 87.482 (86.950-88.015) | 85.054 (84.482-85.627) |
| AUC ROC | 88.956 (88.464-89.458) | 86.316 (85.890-86.831) |
| F1-Score | 62.938 (61.048-64.829) | 62.506(60.765-64.247) |
| Rural | | |
| Precision | 72.449 (67.936-76.962) | 72.619 (68.647-76.592) |
| Sensitivity aka. Recall | 44.306 (40.382-48.230) | 46.329 (42.781-49.877) |
| Specificity | 95.996 (95.240-96.751) | 94.386 (93.457-95.314) |
| Accuracy | 86.069 (84.871-87.268) | 82.697 (81.370-84.325) |
| AUC ROC | 85.900 (84.851-86.829) | 83.551 (82.112-84.905) |
| F1-Score | 54.985 (50.607-59.364) | 56.569 (52.671-60.466) |
