# Supplementary figures and images for: Predicting COVID-19–Related Health Care Resource Utilization Across a Statewide Patient Population: Model Development Study
Source: J Med Internet Res. 2021 Nov 15;23(11):e31337. doi: 10.2196/31337 (PMC8594735; doi:10.2196/31337)

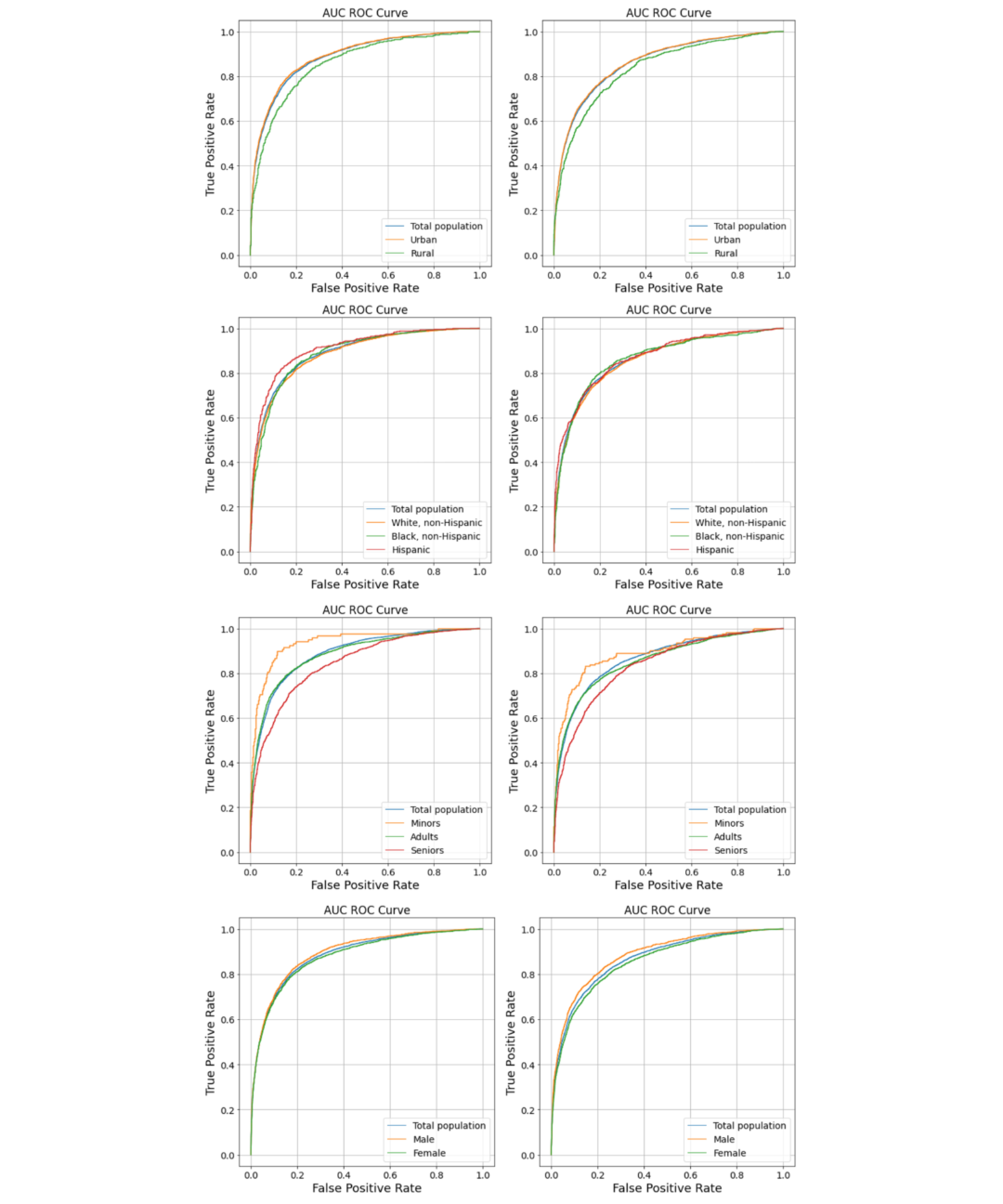

Supplement: Multimedia Appendix 3 [file jmir_v23i11e31337_app3.png]
